# We Built it, But Did They Come: Veterans’ Use of VA Healthcare System-Provided Complementary and Integrative Health Approaches

**DOI:** 10.1007/s11606-022-07889-4

**Published:** 2022-11-30

**Authors:** Stephanie L. Taylor, Hannah M. Gelman, Rian DeFaccio, Jamie Douglas, Matthew J. Hawrilenko, Nathan K. McGinty, Adam Resnick, Nathan C. Tomlanovich, Joy Toyama, Alison M. Whitehead, Benjamin Kligler, Steven B. Zeliadt

**Affiliations:** 1 Center for the Study of Healthcare Innovation, Implementation and Policy, Health Services Research & Development, Greater Los Angeles VA Healthcare System, MC 151, 11301 Wilshire, Bldg. 206, 2nd Floor, Los Angeles, CA 90073 USA; 2 Department of Medicine and Department of Health Policy and Management, UCLA, Los Angeles, CA USA; 3 Center of Innovation for Veteran-Centered and Value-Driven Care, VA Puget Sound Healthcare System, Seattle, WA USA; 4 Integrative Health Coordinating Center, Office of Patient-Centered Care and Cultural Transformation, Veterans Health Administration, Washington, DC USA; 5 Department of Health Services, School of Public Health, University of Washington, Seattle, WA USA

**Keywords:** veteran, complementary and integrative health, complementary and alternative medicine, yoga, meditation

## Abstract

**Background:**

Interest in complementary and integrative health (CIH) approaches, such as meditation, yoga, and acupuncture, continues to grow. The evidence of effectiveness for some CIH approaches has increased in the last decade, especially for pain, with many being recommended in varying degrees in national guidelines. To offer nonpharmacological health management options and meet patient demand, the nation’s largest integrated healthcare system, the Veterans Health Administration (VA), greatly expanded their provision of CIH approaches recently.

**Objective:**

This paper addressed the questions of how many VA patients might use CIH approaches and chiropractic care if they were available at modest to no fee, and would patients with some health conditions or characteristics be more likely than others to use these therapies.

**Design:**

Using electronic medical records, we conducted a national, three-year, retrospective analysis of VA patients’ use of eleven VA-covered therapies: chiropractic care, acupuncture, Battlefield Acupuncture, biofeedback, clinical hypnosis, guided imagery, massage therapy, meditation, Tai Chi/Qigong, and yoga.

**Participants:**

We created a national cohort of veterans using VA healthcare from October 2016–September 2019.

**Key Results:**

Veterans’ use of these approaches increased 70% in three years. By 2019, use was 5.7% among all VA patients, but highest among patients with chronic musculoskeletal pain (13.9%), post-traumatic stress disorder (PTSD; 10.6%), depression (10.4%), anxiety (10.2%), or obesity (7.8%). The approach used varied by age and race/ethnicity, with women being uniformly more likely than men to use each approach. Patients having chronic musculoskeletal pain, obesity, anxiety, depression, or PTSD were more likely than others to use each of the approaches.

**Conclusions:**

Veterans’ use of some approaches rapidly grew recently and was robust, especially among patients most in need. This information might help shape federal/state health policy on the provision of evidence-based CIH approaches and guide other healthcare institutions considering providing them.

## INTRODUCTION

Interest in complementary and integrative health (CIH) approaches for managing wellness and health continues to grow.^1^ These approaches include meditation, yoga, and acupuncture. The evidence of effectiveness ^2–12^ for some CIH approaches for health has increased in the last decade, especially for pain, leading many to be included in the Department of Health and Human Services national pain management strategy^13^ and American College of Physicians’ pain management clinical guidelines.^14^ The search for non-opioid pain management options has further spurred increasing demand for CIH and other nonpharmacological therapies.

The Veterans Health Administration (VA), the nation’s largest integrated healthcare system providing care to over 9 million patients a year, is in the midst of transforming to a Whole Health System of care.^15^ This is a shift from focusing on episodic, disease-centered care to engaging and empowering patients throughout their lives to take charge of their life and health, emphasizing well-being and self-care along with conventional care and CIH approaches. This recent shift, backed by veteran demand^16^ and strong support from Congress and VA leadership^17^, has led to a great interest in implementing CIH programs across the VA.^17,18^ Since 2016, VA has been providing CIH approaches to its patients as part of the medical benefits package. VA has been providing chiropractic care as allopathic treatment since the early 2000s. Our 2017–2018 national survey of VA’s healthcare system showed CIH programs proliferated throughout the VA, although not every CIH approach was available at every VA medical facility at the time.^19^ However, researchers have not yet examined utilization of these approaches in the VA nationally.

As an integrated healthcare system, the VA is in a relatively unique position to examine the question of how much patients might use CIH approaches if they were available at modest-to-no fee. Co-pays for these approaches in the VA are typically either low or non-existent, and are determined by the clinic in which they are located and follow VA business rules. Some veterans using the VA healthcare system are exempt from making co-pays due to their disability rating, income level, or special eligibility factors. Standard healthcare systems might be interested in knowing how much CIH patients might use if the approaches were low/no cost as they consider spending resources to implement CIH programs themselves. The “if we build it, will they come?” question concerns many.

As such, in a research-operations effort, we partnered with the VA office overseeing CIH policy and strategy to conduct the first national examination of the prevalence, trends, and correlates of veterans’ use of VA-covered CIH approaches using a national cohort of VA healthcare system users we created for this purpose. These data may not necessarily generalize to other healthcare systems as veterans most likely have a higher need for CIH therapies than the general population in that they have higher rates of chronic conditions such as pain, anxiety, and depression. The specific populations of other healthcare systems may reflect different needs for CIH therapies.^20,21^ Because of VA’s size, this large expansion in CIH provision also has potential to affect federal and state health policy on CIH provision within any healthcare setting.

## METHODS

We conducted a national retrospective examination of electronic health records (EHRs) in the VA healthcare system to analyze veterans’ use of VA-covered CIH approaches and chiropractic care. The Greater Los Angeles VA’s local IRB declared a quality improvement effort, not research.

### Cohort

We created a national cohort of active VA healthcare users by identifying all unique veterans having at least one VA primary care, mental health, or pain clinic visit for each fiscal year (October to September) between 2016 and 2019. Demographic and clinical characteristics, such as age, were determine based on the first (index) visit date of each fiscal year.

### CIH Approaches

We examined veterans’ use of chiropractic care and all CIH approaches that were covered under the medical benefits package: chiropractic care, acupuncture (traditional and Battlefield Acupuncture), biofeedback, clinical hypnosis, guided imagery, massage therapy, meditation, Tai Chi/Qigong, and yoga (see brief descriptions at the bottom of Table 1). Although the VA considers chiropractic care conventional care, many consider it a CIH approach^22^ so we included it. Battlefield Acupuncture is a protocolized acupuncture treatment currently unique to the VA and military healthcare systems and performed by trained professionals for the purpose of relieving acute and/or chronic pain.^23^

**Table 1:** Three-year trends in veterans’ use of CIH approaches and chiropractic care

Therapy	10/2016–9/2017		10/2017–9/2018		10/2018–9/2019		% change No. of users*	% change No. of visits*
	No. of users	No. of visits	No. of users	No. of visits	No. of users	No. of visits		
Any	177,253	1,567,387	226,539	2,072,023	302,296	2,792,653	70.5%	78.2%
Chiropractic	96,752	805,301	118,704	952,960	159,506	1,224,324	64.9%	52.0%
Acup.-Traditional	67,461	516,857	88,367	689,313	112,826	868,728	67.2%	68.1%
Acup.-BFA	8520	20,474	17,255	43,796	27,990	79,911	228.5%	290.3%
Massage Therapy	21,031	134,908	26,254	248,948	38,582	386,828	83.5%	186.7%
Meditation	4762	18,839	8218	34,079	15,317	60,866	221.7%	223.1%
Yoga	6242	40,659	8899	57,847	14,424	92,163	131.1%	126.7%
Tai Chi/Qigong	2510	17,953	4614	30,024	9806	62,038	290.7%	245.6%
Biofeedback	3065	9778	3619	12,176	3534	12,051	15.3%	23.2%
Guided Imagery	335	788	387	994	1340	3209	300.0%	307.2%
Clinical Hypnosis	686	1830	828	1886	1138	2535	65.9%	38.5%

*Three year change from 10/2016 to 9/2019. *BFA* Battlefield Acupuncture

Acupuncture—one of several techniques that make up the system of care provided by those trained in traditional medicine from China and other Asian countries. Acupuncture may refer to this whole system approach to health care or define the technique of acupuncture treatment. Most frequently we think of acupuncture as the penetration of thin needles into the body at acupuncture points to effect a change

Biofeedback—a process that uses your body’s own signals like heart rate and body temperature to bring about healthy changes

Clinical hypnosis—the process of (a) deliberately triggering a trance state and then (b) utilizing that state to encourage helpful cognitive, emotional, or physical healing responses. A trance is a natural biological state of inner absorption, concentration, and focused attention

Massage therapy—the manipulation of the soft tissues of the human body for therapeutic purposes

Meditation—a practice or technique, often arising from a contemplative tradition, that primarily focuses on training attention regulation processes, with the intent of cultivating general mental well-being and/or specific capacities such as concentration, compassion, or insight. The focus is on training attentional processes, rather than specifically targeting a change in mental contents

Guided imagery—using a series of multi-sensory images designed to trigger specific changes in physiology, emotions, or mental state for the purpose of increasing healing response or unconscious changes. It often begins with a series of relaxation techniques, although this is not always so

Tai Chi—a mind-body exercise combining slow-flowing intentional movements with breathing, awareness, and visualization. Rooted in the Asian traditions of martial arts, Chinese medicine and philosophy, it enhances relaxation, vitality, focus, posture, balance, strength, flexibility, and mood

Qigong—an ancient Chinese healing art, older than, and similar to Tai Chi, with a focus of cultivating the body’s vital energy or qi. It involves the coordination of the breath, posture, awareness, visualization and focused movements. It may be a stationary or moving meditation

Yoga—a mind and body practice with origins in ancient Indian philosophy. The various styles of yoga typically combine physical postures, breathing techniques, and meditation or relaxation

As noted above, we examined all VA-covered CIH approaches, which are provided in the VA in a variety of ways (e.g., Whole Health programs, stand-alone yoga programs or clinics, meditation embedded in psychological therapy). These also include community-based CIH paid for by the VA (e.g., acupuncture, chiropractic care, and massage therapy). To obtain utilization data, we used the VA’s community-based care claims and the EHR for care delivered by VA-based providers (codes detailed elsewhere)^18^.

### Health Conditions

We examined three types of health conditions, which were selected because either particular CIH therapies have demonstrated evidence of effectiveness for them or anecdotal evidence suggests that patients with particular health conditions have been using some CIH therapies, with little documentation of that use.

#### Moderate-to-Severe Chronic Musculoskeletal Pain

We used an algorithm developed by the NIH-DoD-VA Pain Management Collaboratory^24^ which requires patients meet two criteria. First, patients had to have two moderate-to-severe pain severity scores on the numeric rating scale (NRS ≥4) in the year prior to the index visit separated by at least 30 days. Second, they also had to have an ICD10 code related to musculoskeletal pain in the year prior to the index visit, using codes identified by Goulet et al. (2016) ^25^ for back pain; neck pain; limb/extremity pain, joint pain, and arthritic disorders, except gout and other crystal arthropathies or neuropathic arthropathy; fibromyalgia; tension headache; orofacial, ear, and temporomandibular disorder pain; musculoskeletal chest pain; and general musculoskeletal pain.

#### Other Chronic Health Conditions

We identified veterans with cardiovascular disease, diabetes or obesity by the presence of a relevant ICD10 code in the year prior to their index visit, using ICD10 codes based on ICD9 codes in the Elixhauser comorbidity index^26^.

#### Mental Health Conditions

We defined patients as having depression, anxiety, or PTSD as those having a relevant ICD10 code (based on a VA ICD9 diagnosis code list^27^) in the year prior to their index visit.

### Analysis

We first calculated the number of visits patients made between October 2016 and September 2019 and number of unique patients making those visits. We then conducted unadjusted and adjusted analyses (producing relative risk estimates) to examine associations between veterans’ use of the eleven approaches and their health and sociodemographic characteristics. Given the growth in use we observed over 3 years, we conducted these analyses on only the most recent year of our data. We used the Holm-Bonferroni multiple comparison adjustment and conducted analyses with R statistical software version 3.5.3 (R Project for Statistical Computing).

## RESULTS

Of the 5,260,921 veteran patients in our national cohort of VA healthcare system users between October 2018 and September 2019, 302,296 veterans or 5.7% used chiropractic care or any of the ten CIH approaches provided by the VA within VA facilities or VA community care. They also made 2,792,653 visits for these approaches. Chiropractic care (3.0%), traditional acupuncture (2.1%), massage therapy (0.7%), Battlefield Acupuncture (0.5%), meditation (0.3%), and yoga (0.3%) were the most frequently used.

Table 1 shows this utilization represents a substantial increase relative to 2 years prior, in that the number of veteran patients using them increased by 70.5% and the number of visits made increased by 78.2%. The number of users more than doubled for guided imagery, Battlefield Acupuncture, Tai Chi/Qigong, meditation, and yoga, but did not grow as much for the two most frequently used approaches (traditional acupuncture and chiropractic care).

Tables 2 and 3 present the results of univariate analyses to show the demographic characteristics of VA healthcare system patients using the CIH approaches or chiropractic care and also of the entire VA cohort from which they were sampled. They show, for example, that women appeared more likely to use these approaches. At the high end, women accounted for 26.5% of yoga users and, at the low end, 16.8% of chiropractic care users. Also, veterans under 50 years old were more likely to CIH approaches in general relative to older veterans, as they represented 37.2% of patients using the CIH approaches or chiropractic care but only 22.5% of the VA healthcare users. Finally, veterans living in urban areas appeared as likely as other veterans to use CIH therapies and chiropractic care.

**Table 2:** Demographic characteristics of veterans using CIH approaches or chiropractic care: frequencies, Oct. 2018–Sept. 2019, part 1

	All VHA patients	Any activity	Chiropractic	Acup.-Traditional	BFA*	Massage therapy
Overall	5,260,921	302,298	159,507	112,826	27,991	38,582
Gender						
Male	91.0%	82.7%	83.2%	81.5%	80.7%	82.6%
Female	9.0%	17.3%	16.8%	18.5%	19.3%	17.4%
Age						
18–39	13.2%	20.8%	26.1%	16.4%	14.6%	20.0%
40–49	9.5%	16.4%	18.3%	15.7%	14.9%	16.6%
50–59	14.4%	20.3%	19.8%	21.2%	22.4%	20.4%
60–69	22.6%	21.5%	18.9%	22.9%	24.4%	21.0%
70 +	40.3%	21.0%	16.9%	23.7%	23.7%	22.0%
Race*						
White	71.9%	70.4%	73.0%	68.7%	71.6%	71.3%
Black	17.5%	18.1%	14.9%	19.1%	19.8%	15.5%
Asian	1.1%	2.0%	2.1%	2.4%	0.7%	2.4%
NHOPI	0.8%	1.3%	1.5%	1.3%	0.8%	2.0%
AIAN	0.8%	0.9%	1.0%	0.9%	1.0%	0.9%
Unknown	8.0%	7.3%	7.5%	7.5%	6.2%	8.0%
Ethnicity*						
Not Hisp. Latino	90.7%	89.5%	90.0%	89.7%	92.6%	83.8%
Hisp Latino	6.2%	8.3%	7.7%	8.1%	5.5%	13.8%
Unknown	3.1%	2.2%	2.3%	2.2%	1.9%	2.4%
Urban						
Yes	76.7%	78.3%	76.0%	81.6%	75.4%	74.4%
No	22.1%	20.2%	23.2%	17.8%	24.0%	18.0%
Unknown	1.3%	1.5%	0.8%	0.5%	0.5%	7.6%

*NHOPI Native Hawaiian or Other Pacific Islander, *AIAN* American Indian or Alaska Native, *BFA* Battlefield Acupuncture

**Table 3:** Demographic characteristics of veterans using CIH approaches or chiropractic care: frequencies, Oct. 2018–Sept. 2019, part 2

	Meditation	Yoga	Tai Chi/ Qigong	Biofeedback	Guided Imagery	Clinical Hypnosis
Overall	15,317	14,424	9806	3534	1340	1138
Gender						
Male	79.5%	73.5%	79.2%	77.4%	77.5%	79.3%
Female	20.5%	26.5%	20.8%	22.6%	22.5%	20.7%
Age						
18–39	16.3%	16.8%	10.0%	24.1%	13.6%	13.4%
40–49	14.3%	15.2%	11.8%	17.9%	13.7%	13.8%
50–59	23.1%	22.8%	21.2%	23.2%	21.8%	24.1%
60–69	25.7%	24.9%	29.8%	20.8%	27.2%	28.3%
70 +	20.6%	20.3%	27.2%	14.1%	23.7%	20.4%
Race*						
White	65.2%	62.8%	62.6%	64.3%	63.0%	67.4%
Black	26.0%	27.9%	28.6%	27.2%	28.4%	26.0%
Asian	0.9%	1.3%	1.1%	1.1%	0.7%	0.2%
NHOPI	0.8%	0.8%	0.8%	0.8%	0.7%	0.8%
AIAN	0.9%	0.9%	0.8%	0.6%	0.9%	1.1%
Unknown	6.3%	6.2%	6.0%	5.9%	6.2%	4.6%
Ethnicity*						
Not Hisp. L.	90.6%	91.0%	92.2%	90.7%	92.1%	93.6%
Hisp L.	7.7%	7.2%	6.2%	7.7%	6.3%	5.3%
Unknown	1.7%	1.8%	1.6%	1.6%	1.6%	1.1%

*NHOPI Native Hawaiian or Other Pacific Islander, *AIAN* American Indian or Alaska Native, *HL* Hispanic or Latino

We then examined CIH use among veteran patients with common health conditions that might be affected by a particular CIH approach. We first determined the percentages of all VA healthcare system users from October 2018 to September 2019 having each condition. We found 60.1% of veterans had cardiovascular disease, 24.3% had diabetes, 22.7% had chronic musculoskeletal pain, 18% had PTSD, 17.4% were obese, 17.2% had depression, and 13.2% had anxiety. Table 4 presents the results of univariate analyses to show the percentage of all VA patients with a particular health condition using CIH approaches from October 2018 to September 2019. Although 5.7% of veterans used any of the approaches we examined, the table shows that more veterans used these approaches if they had one of these health conditions. Specifically, 13.9% of veterans with chronic musculoskeletal pain used any of them, as did 10.6% of those with PTSD, 10.4% of those with depression, 10.2% of those with anxiety, and 7.8% of those with obesity.

**Table 4:** Percent of veterans with specific health conditions who used CIH approaches or chiropractic care: frequencies, Oct. 2018–Sept. 2019 (*n*= 5,260,920)

	Any activity	Chiropractic	Acup.-Trad.	BFA*	Massage therapy	
Chronic musculoskeletal pain	13.9%	6.8%	6%	1.6%	1.8%	
Anxiety	10.2%	5.1%	3.8%	1.1%	1.2%	
Depression	10.4%	4.8%	4.1%	1.2%	1.2%	
PTSD*	10.6%	5.3%	4.1%	1.0%	1.3%	
CVD*	4.9%	2.3%	1.9%	0.5%	0.6%	
Diabetes	5.1%	2.3%	2.1%	0.6%	0.7%	
Obesity	7.8%	3.9%	3%	0.8%	1.0%	
	Meditation	Yoga	Tai Chi/ Qigong	Biofeedback	Guided Imagery	Clinical Hypnosis
Chronic musculoskeletal pain	0.7%	0.7%	0.5%	0.2%	0.1%	0.1%
Anxiety	0.8%	0.7%	0.4%	0.2%	0.1%	0.1%
Depression	0.8%	0.8%	0.5%	0.2%	0.1%	0.1%
PTSD*	0.8%	0.7%	0.4%	0.2%	0.1%	0.1%
CVD*	0.3%	0.3%	0.2%	0.1%	< 0.1%	< 0.1%
Diabetes	0.3%	0.3%	0.2%	0.1%	< 0.1%	< 0.1%
Obesity	0.5%	0.5%	0.4%	0.1%	0.1%	< 0.1%

*PTSD post-traumatic stress disorder, *CVD* cardiovascular disease (includes hypertension), *BFA* Battlefield Acupuncture

Tables 5 and 6 show the results of multivariate analyses examining associations between using (1) any of the approaches or (2) each specific approach and veterans’ characteristics or health conditions. The results show, for example, that women were more likely than men to use each of the approaches; they were 35% more likely than men to use any approach and 25% more likely to use chiropractic care. Also, as veterans got older, they appeared less likely to use these approaches in general. However, older veterans were more likely than younger to use some specific approaches. For example, those ages 40–50 were more likely than those ages 19–39 to use eight of the eleven approaches. Among older veterans, those ages 50–59 appeared more likely than others to use meditation and 60–69-year-old veterans appeared more likely to use Tai Chi/Qigong. Urban dwellers also appeared more likely than rural dwellers to use eight of the eleven approaches. When examining racial/ethnic patterns, we found that Blacks were more likely than Whites to use yoga, Tai Chi/Qigong, and guided imagery, while Hispanic Latinos were more likely than those not identifying as Hispanic Latino to use acupuncture, massage therapy, and meditation.

**Table 5:** Multivariate predictors of veterans’ use of CIH approaches or chiropractic care, Oct. 2018–Sept. 2019: part 1 (*n*=5,260,921)

	Any activity	Chiropractic care	Acupuncture-traditional	Battlefield acupuncture	Massage therapy	Meditation
	RR (CI)	RR (CI)	RR (CI)	RR (CI)	RR (CI)	RR (CI)
Gender						
Male	--	--	--	--	--	--
Female	1.35(1.34, 1.36)	1.25(1.23, 1.26)	1.52(1.49, 1.54)	1.61(1.56, 1.66)	1.44(1.40, 1.48)	1.49(1.42, 1.55)
Age						
18–39	--	--	--	--	--	--
40–49	1.03(1.02, 1.04)	0.94(0.92, 0.95)	1.23(1.21, 1.26)	1.23(1.18, 1.29)	1.07(1.04, 1.11)	1.18(1.11, 1.25)
50–59	0.88(0.87, 0.89)	0.69(0.68, 0.70)	1.15(1.13, 1.18)	1.22(1.17, 1.27)	0.91(0.88, 0.94)	1.43(1.35, 1.51)
60–69	0.67(0.67, 0.68)	0.45(0.45, 0.46)	0.94(0.92, 0.96)	0.99(0.95, 1.04)	0.67(0.65, 0.69)	1.34(1.27, 1.42)
70 +	0.47(0.46, 0.47)	0.27(0.26, 0.27)	0.75(0.73, 0.76)	0.76(0.72, 0.79)	0.49(0.47, 0.51)	0.87(0.81, 0.92)
Race						
White	--	--	--	--	--	--
Black	0.74(0.74, 0.75)	0.60(0.59, 0.61)	0.76(0.74, 0.77)	0.75(0.72, 0.77)	0.65(0.63, 0.67)	1.08(1.04, 1.12)
Other	1.28(1.26, 1.30)	1.31(1.28, 1.34)	1.49(1.45, 1.53)	0.77(0.72, 0.84)	1.48(1.42, 1.55)	0.86(0.78, 0.95)
Unknown	0.96(0.94, 0.97)	0.93(0.91, 0.95)	1.00(0.98, 1.03)	0.87(0.82, 0.92)	1.01(0.96, 1.05)	0.90(0.84, 0.98)
Ethnicity						
Not Hispanic Latino	--	--	--	--	--	--
Hispanic Latino	0.99(0.97, 1.00)	0.89(0.88, 0.91)	1.06(1.03, 1.08)	0.74(0.70, 0.78)	1.19(1.15, 1.23)	1.08(1.01, 1.15)
Unknown	0.85(0.83, 0.88)	0.85(0.82, 0.89)	0.85(0.82, 0.89)	0.82(0.74, 0.91)	0.89(0.82, 0.96)	0.82(0.71, 0.94)
Urban						
Yes	--	--	--	--	--	--
No	1.02(1.01, 1.03)	1.21(1.19, 1.22)	0.86(0.85, 0.87)	1.22(1.19, 1.26)	0.95(0.93, 0.98)	0.69(0.66, 0.73)
Unknown	1.20(1.17, 1.24)	0.70(0.66, 0.74)	0.39(0.36, 0.42)	0.52(0.44, 0.62)	5.78(5.55, 6.02)	0.21(0.15, 0.28)
Chronic Musculoskeletal Pain						
No	--	--	--	--	--	--
Yes	3.59(3.56, 3.61)	3.25(3.22, 3.28)	5.13(5.06, 5.20)	5.61(5.46, 5.76)	3.91(3.82, 3.99)	2.25(2.17, 2.32)
Anxiety						
No	--	--	--	--	--	--
Yes	1.17(1.16, 1.18)	1.08(1.07, 1.10)	1.17(1.16, 1.19)	1.36(1.32, 1.40)	1.10(1.07, 1.13)	1.90(1.83, 1.98)
Depression						
No	--	--	--	--	--	--
Yes	1.20(1.19, 1.21)	1.03(1.02, 1.04)	1.23(1.21, 1.25)	1.49(1.45, 1.53)	1.07(1.04, 1.10)	2.19(2.11, 2.28)
PTSD						
No	--	--	--	--	--	--
Yes	1.37(1.36, 1.38)	1.25(1.23, 1.26)	1.43(1.41, 1.45)	1.40(1.36, 1.44)	1.34(1.31, 1.37)	2.17(2.09, 2.25)
Diabetes						
No	--	--	--	--	--	--
Yes	0.98(0.98, 0.99)	0.94(0.93, 0.96)	0.98(0.96, 0.99)	1.05(1.03, 1.09)	1.01(0.99, 1.04)	1.09(1.05, 1.14)
Obesity						
No	--	--	--	--	--	--
Yes	1.17(1.16, 1.18)	1.12(1.10, 1.13)	1.17(1.16, 1.19)	1.28(1.24, 1.31)	1.17(1.14, 1.20)	1.52(1.46, 1.57)
Cardiovascular Disease						
No	--	--	--	--	--	--
Yes	0.90(0.89, 0.91)	0.87(0.86, 0.88)	0.89(0.88, 0.90)	1.02(0.99, 1.05)	0.90(0.88, 0.92)	1.00(0.97, 1.04)

*RR* rate ratio, *CI* 95% confidence interval

**Table 6:** Predictors of veterans’ use of CIH approaches or chiropractic care, Oct. 2018–Sept. 2019: part 2 (*n*=5,260,921)

	Yoga	Tai Chi/Qigong	Biofeedback	Guided Imagery	Clinical Hypnosis
	RR (CI)	RR (CI)	RR (CI)	RR (CI)	RR (CI)
Gender	***	***	***	***	***
Male	--	--	--	--	--
Female	2.10(2.02, 2.19)	1.79(1.7, 1.89)	1.37(1.26, 1.49)	1.79(1.56, 2.06)	1.56(1.34, 1.83)
Age					
18–39	--	--	--	--	--
40–49	1.20(1.13, 1.27)	1.49(1.37, 1.62)	1.02(0.92, 1.14)	1.24(1.01, 1.53)	1.33(1.06, 1.67)
50–59	1.38(1.31, 1.46)	1.98(1.83, 2.14)	1.03(0.93, 1.14)	1.41(1.16, 1.72)	1.67(1.36, 2.06)
60–69	1.36(1.29, 1.44)	2.42(2.23, 2.62)	0.81(0.73, 0.91)	1.52(1.24, 1.86)	1.63(1.31, 2.02)
70 +	0.95(0.89, 1.01)	1.89(1.73, 2.06)	0.46(0.40, 0.52)	1.15(0.92, 1.44)	0.96(0.76, 1.22)
Race					
White	--	--	--	--	--
Black	1.11(1.07, 1.16)	1.28(1.22, 1.34)	1.08(0.99, 1.17)	1.23(1.08, 1.41)	1.01(0.87, 1.16)
Other	1.02(0.93, 1.12)	1.05(0.93, 1.18)	0.78(0.63, 0.96)	0.88(0.61, 1.25)	0.67(0.44, 1.01)
Unknown	0.91(0.84, 0.98)	0.98(0.88, 1.08)	0.84(0.71, 0.99)	1.03(0.79, 1.33)	0.72(0.52, 0.99)
Ethnicity					
Not Hispanic Latino	--	--	--	--	--
Hispanic Latino	0.95(0.89, 1.02)	1.00(0.92, 1.09)	0.94(0.83, 1.08)	0.88(0.70, 1.10)	0.72(0.54, 0.96)
Unknown	0.84(0.72, 0.97)	0.72(0.6, 0.87)	0.80(0.59, 1.09)	0.68(0.41, 1.11)	0.64(0.34, 1.20)
Urban					
Yes	--	--	--	--	--
No	0.54(0.51, 0.57)	0.72(0.68, 0.76)	0.53(0.48, 0.59)	0.94(0.81, 1.09)	0.85(0.73, 0.99)
Unknown	0.54(0.44, 0.66)	0.07(0.04, 0.14)	0.21(0.11, 0.4)	0.88(0.51, 1.53)	0.61(0.27, 1.36)
Chronic Musculoskeletal pain					
No	--	--	--	--	--
Yes	2.54(2.45, 2.63)	2.63(2.52, 2.75)	3.02(2.81, 3.25)	3.20(2.84, 3.61)	3.45(3.03, 3.93)
Anxiety					
No	--	--	--	--	--
Yes	1.51(1.46, 1.58)	1.51(1.44, 1.59)	2.51(2.32, 2.71)	1.73(1.52, 1.97)	1.69(1.46, 1.95)
Depression					
No	--	--	--	--	--
Yes	1.96(1.88, 2.04)	1.85(1.76, 1.94)	1.87(1.73, 2.03)	2.12(1.86, 2.42)	1.93(1.67, 2.22)
PTSD					
No	--	--	--	--	--
Yes	2.32(2.24, 2.41)	2.02(1.93, 2.12)	2.38(2.21, 2.56)	1.87(1.65, 2.12)	1.88(1.64, 2.15)
Diabetes					
No	--	--	--	--	--
Yes	0.96(0.92, 1.00)	1.08(1.03, 1.13)	0.98(0.90, 1.07)	1.03(0.91, 1.18)	1.13(0.98, 1.30)
Obesity					
No	--	--	--	--	--
Yes	1.70(1.64, 1.76)	2.03(1.95, 2.12)	1.21(1.12, 1.31)	1.75(1.56, 1.97)	1.34(1.17, 1.53)
Cardiovascular Disease					
No	--	--	--	--	--
Yes	0.94(0.90, 0.98)	0.91(0.87, 0.96)	0.94(0.87, 1.02)	1.04(0.91, 1.19)	0.89(0.77, 1.02)

*RR* relative risk, *CI* 95% confidence interval

When examining health conditions, veterans with chronic musculoskeletal pain, obesity, anxiety, depression, or PTSD were more likely than others without those conditions to use each of the eleven approaches, while veterans with cardiovascular disease were less likely. Finally, veterans with diabetes were more likely than those without to use Battlefield Acupuncture, meditation, yoga, and Tai Chi/Qigong.

## DISCUSSION

We conducted the first national examination of the prevalence and correlates of veterans’ use of ten CIH approaches and chiropractic care provided within VA facilities or VA-covered community care, using a national cohort of VA healthcare system users that we created. The interest in using these approaches for well-being and health is strong^16^, and the VA healthcare system has been rapidly implementing CIH programs to meet that interest^17,19^.

Our analyses of the national cohort of VA healthcare system users showed that almost 6% of veterans used one or more of the eleven approaches during October 2018–September 2019, with traditional acupuncture and chiropractic care being the most frequently used. Our study does not count all the yoga, meditation, and Tai Chi/Qigong that veterans did on their own at home or in community-based classes not covered by the VA, nor does it count visits to other practitioners such as acupuncturists or chiropractors that veterans used on their own without going through the VA. As our earlier survey of veterans show, many choose to receive some care entirely outside the VA system or at VA-funded community care.^16^ Claims for that care are often delayed, so we intentionally waited several months for that data to enter the VA, resulting in our dataset being fairly complete. For these reasons, our study represents only a fraction of the CIH approaches that veterans are using.

When looking at the subpopulations most in need, we found that even more veterans used chiropractic care or CIH approaches. Specifically, almost 14% of veterans with chronic musculoskeletal pain, and about 10% of those with depression, PTSD, or anxiety, used any of the approaches we examined.

To put our VA-provided CIH use percentages into perspective, the most frequently used CIH activity among the *general* population in 2017 was yoga (14.3% used it anywhere—home, community, or healthcare system), followed by meditation (14.2%), and chiropractic care (10.3%), according to the most recent national survey conducted among the general population, the CDC’s NHIS.^1^ It is difficult to neatly compare veterans’ and the general populations’ use for two reasons. Not only do the two analyses examine different CIH-based venues (veteran survey of VA-provided CIH versus NHIS survey of CIH done anywhere), but veterans most likely have a higher need for CIH therapies than the general population in that they have higher rates of chronic conditions such as pain, anxiety, and depression.^20,21^

We also showed veterans use of any of the eleven approaches increased over 70% from 2 years prior. When looking at each therapy individually, we found the number of veteran users more than doubled for half of the therapies. Chiropractic care might not have grown more than it did because the VA has been providing it care as allopathic treatment for almost a decade, so it is natural that the levels of use would be higher and the 3-year growth rate lower than other the therapies.

This remarkable growth might represent increased interest, as the VA is increasing its efforts to inform providers and patients about the availability and effectiveness of their CIH programs. Equally likely, this growth represents increased availability at the VA, as VA medical facilities continue to rapidly implement CIH programs over the past several years. Our 2018–2019 national survey of CIH provision among all VA medical centers showed wide variation in CIH program availability at the time, but most facilities offered a few CIH approaches.^19^ As such, it is likely that this growth in CIH use will continue. As healthcare pivoted to telehealth during the COVID-19 pandemic^28^, it will be interesting to see if the new the tele-meditation, -yoga, -Tai Chi, and -Qigong programs starting to proliferate throughout the VA will lead to more veterans using CIH therapies or use it more often, or if patients will only substitute in-person classes for virtual classes resulting in a net zero gain in users and visits.

We found women were more likely than men to use each approach, which agrees with the CDC’s NHIS survey of the general population.^1^ We also found that veterans’ use of CIH varied by age, with veterans ages 40–50 being more likely than younger veterans and veterans 70 and older being more likely than others to use several approaches. This pattern differs slightly from that among the general population survey, where those over 70 used fewer CIH approaches.

It is unsurprising that urban dwellers appeared more likely to use eight approaches, as they tend to live nearer VA medical facilities or likely have more community-based CIH options. However, this pattern might shift with the availability of tele-CIH, as telehealth might reduce geographic disparities in access. We also found interesting racial/ethnic patterns, in that Blacks were more likely than Whites to use half the CIH approaches, while Hispanic Latinos were more likely to use acupuncture, massage, and meditation. This counters the 2017 NHIS findings among the general population that non-Hispanic Whites were more likely to use several CIH approaches.

Our study has some limitations. First, as with any examination of medical record data, the data are only as good as patients’ use of CIH are documented in medical records. VA’s documentation of care tends to be accurate because documentation drives VA medical facilities’ reimbursement, given the VA is an integrated healthcare system. Also, our results do not easily translate to the general population, given the VA anecdotally offers more CIH approaches than other healthcare systems. However, our population is similar to the general US population having chronic conditions. Finally, as noted earlier, veterans’ actual use of CIH approaches is believed to be higher, when considering what they use on their own, as our earlier survey data shows.^16^

Our results have implications for the VA and other institutions’ provision of CIH approaches for patients, as well as policy implications. The gap between veterans’ use of CIH approaches documented in the VA electronic health records and what they report using in our earlier survey^16^ (which include use of CIH approaches at home, the community or at the VA) suggests a continued need to expand the availability of CIH approaches within the VA. Also, the substantial growth in utilization over 3 years could demonstrate a growth in CIH provision or could reflect patients’ or providers’ changing beliefs about the effectiveness of CIH approaches or their increased awareness of CIH availability. Our results might guide other healthcare systems’ decisions about whether or not to offer these approaches as standard of care. Finally, this information also might provide support federal or state CIH reimbursement policies because it shows the large volume of patients in need who use this type of care.

## CONCLUSIONS

As the VA healthcare system implements CIH programs throughout the nation, veterans’ use of VA-covered CIH approaches rapidly grew and was higher among populations with chronic musculoskeletal pain, depression, PTSD, and anxiety. Many of the CIH utilization patterns we demonstrated were similar to those among the general population, while others were dissimilar. These results might be helpful to other healthcare systems considering providing their constituents with these approaches.

## Declarations

### Disclaimer

The views in this paper are the views of the authors and do not represent the views of the Department of Veterans Affairs or the US Government.
